# Reconstruction of the evolutionary landscape of biological processes involved in the early stages of the metastatic cascade

**DOI:** 10.1590/1678-4685-GMB-2025-0197

**Published:** 2026-06-29

**Authors:** Gleison M. Azevedo, Epitácio Farias, João Vitor F. Cavalcante, Rafaella S. Ferraz, Bruno William, Diego M. Coelho, Rodrigo J.S. Dalmolin

**Affiliations:** 1 Universidade Federal do Rio Grande do Norte, Instituto Metrópole Digital (IMD), Centro Multiusuário de Bioinformática (BioME), Natal, RN, Brazil.; 2 Universidade Federal do Pará, Instituto de Ciências Biológicas, Laboratório de Genética Médica e Humana, Belém, PA, Brazil.; 3 Universidade Federal do Rio Grande do Norte, Centro de Biociências, Departamento de Bioquímica, Natal, RN, Brazil.

**Keywords:** Metastasis, evolution, systems biology, bioinformatics, orthology

## Abstract

Metastasis is not a *de novo* functional module innovation but rather the pathological redeployment of deeply conserved biological programs. Here, we reconstruct the evolutionary landscape of biological functions involved in the early stages of the metastatic cascade, including cell adhesion, extracellular matrix organization (ECM), regulation of metallopeptidase activity, cell junction organization, epithelial-mesenchymal transition (EMT), and cellular extravasation alongside the physiological constraints that suppress them (Metastasis Suppressor Genes). Using phyletic pattern reconstruction of 787 orthologs across 473 Clusters of Orthologous Groups with the Bridge algorithm, we identified a divergence between the evolutionary timelines of metastasis-enabling programs and metastasis suppressors. Our results indicate that the molecular systems associated with the structural capacity for tumor dissemination are evolutionary ancient. ECM organization traces to the Human-Discoba last common ancestor, EMT to Ctenophora, cell adhesion to Sauropsida, and cellular extravasation to Actinopterygii. In contrast, mechanisms responsible for suppressing genomically unstable cells during continuous tissue renewal emerged later, peaking in the vertebrate lineage. These findings support the Serial Atavism Model and suggest that metastasis arises from the progressive erosion of recently evolved regulatory constraints, allowing the reactivation of ancestral cellular programs that predate complex multicellularity.

## Introduction

Metastasis, a hallmark of aggressive cancers, represents a complex, multiphasic process in which primary tumor cells disseminate to distant tissues, forming secondary tumor *foci*. This biological cascade involves distinct cellular events: invasion of adjacent tissues, intravasation into the circulatory system, survival in circulation, extravasation, and colonization of new organs (Pachmayr et al., 2017; Welch and Hurst, 2019; Fares et al., 2020).

Many processes central to metastasis, including cell adhesion and epithelial-mesenchymal transition (EMT), have orthologs in evolutionarily distant lineages, extending even to unicellular organisms (Olson, 2006; Castro et al., 2008, 2009; Robert, 2010; Chen et al., 2015; Viscardi et al., 2021; Feuermann et al., 2025). This conservation suggests that the metastatic potential of cancer cells is not an exclusively pathological innovation but rather a consequence of genetic programs that were established early in eukaryotic evolution (Chen *et al*., 2015). 

Identifying the origin of these orthologs can offer insights into how metastasis-related biological processes emerged as a biological phenomenon. Early phylogenetic studies, such as those by Chen and collaborators (2015), traced the emergence of human protein-coding orthologs across major clades and revealed a significant overrepresentation of cancer-driver orthologs at the dawn of multicellularity. While this highlighted the ancient roots of oncogenesis, modern evolutionary theories suggest that metastatic progression is more than just a single reversion to a unicellular state (Lineweaver et al., 2021; Butler et al., 2025; Ivanov et al., 2025).

The Serial Atavism Model (SAM) proposes that cancer progression is a sequence of atavistic reversions, losing evolutionary traits in the reverse order of their acquisition (Lineweaver et al., 2021). Under this perspective, the most recently evolved homeostatic traits, such as strict tissue boundaries and the constant cellular renewal mechanisms unique to vertebrates, are the first constraints to be deactivated. This progressive loss sequentially unlocks older, more basal evolutionary behaviors, shifting from metazoan cellular mobility down to unconstrained unicellular proliferation. This atavistic regression is not only morphological but also deeply metabolic and genomic. For instance, the dysregulation of energy metabolism (e.g., the Warburg effect) (Liberti and Locasale, 2016) is increasingly understood as an atavistic reversion to an archaic, pre-oxygen era state that confers a strong survival advantage to proliferating cells (Fanchon and Stéphanou, 2024). Similarly, phylostratigraphic analyses of human co-expression networks, such as those in ovarian cancer, demonstrate that tumors exploit an imbalance between ancient, conserved “*caretaker*” genes and more recently evolved “*gatekeeper*” signaling genes (Zhang et al., 2019).

Exploring this hypothesis requires mapping the evolutionary landscape of these biological programs, a task that has historically been computationally intensive. However, recent advances using the phyletic patterns of orthologs have proven highly effective for reconstructing the history of complex traits. For instance, tools like GeneBridge (Campos et al., 2024), paired with the STRING framework, have successfully traced the massive emergence of microprotein orthologs and major neurotransmitter systems (Viscardi et al., 2021; Fesenko et al., 2025).

Building on this approach, our study maps the macro-evolutionary patterns of the key biological processes involved in the early stages of the metastatic cascade, classified as cell adhesion, ECM organization, EMT, regulation of metallopeptidase activit, cell junction organization, and extravasation. Furthermore, to analyze our data within the framework of the SAM, we trace the emergence of Metastasis Suppressor orthologs, the critical physiological constraints responsible for suppressing these early behaviors. By reconstructing this evolutionary scenario and pinpointing the massive emergence of these specific traits, we aim to clarify the genetic and evolutionary foundations of metastasis. Ultimately, this orthologs’ origin perspective establishes a quantitative framework to understand how the gradual deactivation of these evolutionary regulatory layers fuels tumor progression.

## Methods

**Figure 1 -:**
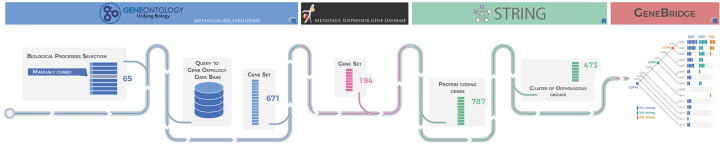
Methodology workflow overview. Biological processes related to metastasis were selected from the GO database based on direct experimental evidence. Genes associated with each process were retrieved via the GO API, and metastasis suppressor genes (MSGs) from the Metastasis Suppressor Genes database. The STRING database was then used to map these genes to STRING IDs, followed by filtering to retain only genes annotated with at least one Cluster of Orthologous Genes (COG) category. Subsequent analyses included pleiotropy assessment, gene rooting using the Last Common Ancestor (LCA) approach via GeneBridge, and temporal functional enrichment estimation across evolutionary clades.

This study investigated the evolution of the early stages of the metastatic cascade biological processes, as detailed in Figure 1. Biological processes were selected from the Gene Ontology (GO) database using AmiGO 2, focusing on experimentally supported terms. Associated genes were then identified for evolutionary analyses, employing computational tools to determine the most probable evolutionary root and explore patterns across clades.

### Early metastasis biological process selection

Biological processes associated with the early stages of the metastatic cascade, including local invasion, intravasation, survival in the circulatory system, and extravasation (Fidler, 2003; Valastyan and Weinberg, 2011), were curated using AmiGO 2 (v. 2.5.17) (Carbon et al., 2009). By navigating the GO hierarchy, we filtered the biological processes based on literature evidence. Nodes representing biological processes unrelated to tumor dissemination were excluded, isolating the functional sub-networks fundamental to the metastatic cascade. While these retained biological processes natively govern physiological homeostasis (e.g., wound healing and embryonic development), they represent the exact modular toolkit pathologically co-opted during early metastasis, yielding a curated set of 65 biological processes from which the associated genes were extracted (Table 1).

**Table 1 -:** Curated list of the 65 biological processes fundamental to the early stages of the metastatic cascade. The table presents biological process signatures, their respective Gene Ontology identifiers (GO IDs), and descriptions.

Signature	GO_id	Description
cellular extravasation	GO:0002691	regulation of cellular extravasation
cellular extravasation	GO:0002692	negative regulation of cellular extravasation
cellular extravasation	GO:0002693	positive regulation of cellular extravasation
cell junction organization	GO:0002934	desmosome organization
cell junction organization	GO:0007043	cell-cell junction assembly
cell junction organization	GO:0007044	cell-substrate junction assembly
cell adhesion	GO:0007159	leukocyte cell-cell adhesion
cell adhesion	GO:0007160	cell-matrix adhesion
extracellular matrix organization	GO:0010715	regulation of extracellular matrix disassembly
extracellular matrix organization	GO:0010716	negative regulation of extracellular matrix disassembly
epithelial to mesenchymal transition	GO:0010717	regulation of epithelial to mesenchymal transition
epithelial to mesenchymal transition	GO:0010718	positive regulation of epithelial to mesenchymal transition
epithelial to mesenchymal transition	GO:0010719	negative regulation of epithelial to mesenchymal transition
cell adhesion	GO:0010810	regulation of cell-substrate adhesion
cell adhesion	GO:0010811	positive regulation of cell-substrate adhesion
cell adhesion	GO:0010812	negative regulation of cell-substrate adhesion
cell adhesion	GO:0022407	regulation of cell-cell adhesion
cell adhesion	GO:0022408	negative regulation of cell-cell adhesion
cell adhesion	GO:0022409	positive regulation of cell-cell adhesion
extracellular matrix organization	GO:0030199	collagen fibril organization
cell adhesion	GO:0033627	cell adhesion mediated by integrin
cell adhesion	GO:0033631	cell-cell adhesion mediated by integrin
cell adhesion	GO:0034110	regulation of homotypic cell-cell adhesion
cell adhesion	GO:0034111	negative regulation of homotypic cell-cell adhesion
cell adhesion	GO:0034112	positive regulation of homotypic cell-cell adhesion
cell adhesion	GO:0034114	regulation of heterotypic cell-cell adhesion
cell adhesion	GO:0034115	negative regulation of heterotypic cell-cell adhesion
cell adhesion	GO:0034116	positive regulation of heterotypic cell-cell adhesion
cell adhesion	GO:0098631	cell adhesion mediator activity
cell adhesion	GO:0098632	cell-cell adhesion mediator activity
cell adhesion	GO:0098635	protein complex involved in cell-cell adhesion
cell adhesion	GO:0098636	protein complex involved in cell adhesion
cell junction organization	GO:0034332	adherens junction organization
cell adhesion	GO:0034446	substrate adhesion-dependent cell spreading
extracellular matrix organization	GO:0034769	basement membrane disassembly
cellular extravasation	GO:0035696	monocyte extravasation
cell adhesion	GO:0044331	cell-cell adhesion mediated by cadherin
cell junction organization	GO:0045217	cell-cell junction maintenance
extracellular matrix organization	GO:0048251	elastic fiber assembly
cellular extravasation	GO:0050901	leukocyte tethering or rolling
cellular extravasation	GO:0050904	diapedesis
extracellular matrix organization	GO:0070831	basement membrane assembly
cell adhesion	GO:0071603	endothelial cell-cell adhesion
cellular extravasation	GO:0072672	neutrophil extravasation
cellular extravasation	GO:0072683	T cell extravasation
extracellular matrix organization	GO:0085029	extracellular matrix assembly
extracellular matrix organization	GO:0090091	positive regulation of extracellular matrix disassembly
cell adhesion	GO:0098742	cell-cell adhesion via plasma-membrane adhesion molecules
extracellular matrix organization	GO:0110011	regulation of basement membrane organization
cell junction organization	GO:0120193	tight junction organization
cell junction organization	GO:0150116	regulation of cell-substrate junction organization
cell junction organization	GO:0150117	positive regulation of cell-substrate junction organization
cell junction organization	GO:0150118	negative regulation of cell-substrate junction organization
cell junction organization	GO:0150146	cell junction disassembly
cell junction organization	GO:0150147	cell-cell junction disassembly
extracellular matrix organization	GO:1901201	regulation of extracellular matrix assembly
extracellular matrix organization	GO:1901202	negative regulation of extracellular matrix assembly
extracellular matrix organization	GO:1901203	positive regulation of extracellular matrix assembly
cell junction organization	GO:1901888	regulation of cell junction assembly
cell junction organization	GO:1901889	negative regulation of cell junction assembly
cell junction organization	GO:1901890	positive regulation of cell junction assembly
extracellular matrix organization	GO:1903053	regulation of extracellular matrix organization
extracellular matrix organization	GO:1903054	negative regulation of extracellular matrix organization
extracellular matrix organization	GO:1903055	positive regulation of extracellular matrix organization
regulation of metallopeptidase activity	GO:1905048	regulation of metallopeptidase activity

For the purpose of this study, these biological processes will be referred to by their parent terms, namely: cell adhesion, ECM organization, regulation of metallopeptidase activity, cell junction organization, cellular extravasation, and EMT. The curated 65 biological processes were used to query the AmiGO 2 database, which returned 671 unique genes in total. To distinguish between genes with broad biological roles and those specialized in each biological process, orthologs were further filtered into exclusive (associated with exactly one of the six metastasis-related processes) and pleiotropic (associated with more than one process) subsets. A separate hypergeometric test was conducted on the exclusive genes to verify evolutionary rooting patterns independently (Figure S1).

### Metastasis suppressor genes selection

To investigate the evolution of physiological constraints against metastasis, we mapped the emergence of Metastasis Suppressor Genes (MSG). The MSGs were selected from the Metastasis Suppressor Genes database, an evidence-based knowledgebase of metastasis suppressors (Zhao et al., 2015). This curated list initially comprised 194 unique MSGs.

### Evolutionary rooting analysis

To identify the evolutionary history of the early metastasis-related and the metastasis suppressors orthologs, an evolutionary rooting analysis was performed using GeneBridge Bioconductor R package (v. 0.99.2) (Campos et al., 2024). The Bridge algorithm was used to infer the points of probable origin, representing the Last Common Ancestor (LCA) between each clade and Homo sapiens, for genes within specific Orthologous Groups (OGs). The evolutionary inference was conducted over a comprehensive 476-eukaryote phylogenetic tree from STRING v.11 (Szklarczyk et al., 2019; Viscardi et al., 2021).

Of the 822 unique metastasis-associated genes and MSGs, 787 protein-coding genes annotated with at least one Cluster of Orthologous Groups (COG) identifier (Tatusov et al., 2003) via the geneplast.data package (v0.99.9) were retained. The rooting assignment for each OG was computed using *runBridge* with a penalty of 2 and a threshold of 0.5. To assess the statistical significance of each inferred root against a null distribution of random phyletic patterns, a permutation procedure *runPermutation* was executed with 1,000 iterations and a strict filtering threshold (*stringent* = TRUE).

### Statistical analysis

We employed a dual-track statistical framework to characterize the distinct evolutionary patterns of metastasis promoters and suppressors. For the metastasis promoter track (early metastasis-related orthologs), we conducted a two-step statistical mapping. First, to identify clades showing a significant increase in ortholog emergence, we applied a modified z-score based on the Median Absolute Deviation (MAD-z-score), providing robustness against the inherently skewed nature of ortholog emergence across the phylogenetic tree. Second, for the orthologs originating in each significant outlier clade, we applied a standard hypergeometric test to assess which of the 65 biological processes were statistically over-represented (using total orthologs, clade-specific orthologs, and specific functional intersections).

Conversely, for the metastasis suppressor track (MSG orthologs), we assessed the progressive acquisition of physiological barriers over evolution through a cumulative functional enrichment analysis for GO Biological Process (GO BP) and KEGG pathways. Gene SYMBOLs were converted to ENTREZ IDs using the org.Hs.eg.db annotation package. We applied a cumulative hypergeometric testing framework spanning the evolutionary clades using the clusterProfiler Bioconductor package (Yu, 2024).

For all statistical tests across both analytical tracks, significance was defined using a p-value and q-value cutoff of < 0.05, with values adjusted for multiple testing via the Benjamini-Hochberg (BH) method, computed utilizing the native *phyper* function in R (v4.4.2) alongside clusterProfiler native metrics.

### Computational analysis environment

All analyses were performed in the R environment (v4.4.2) using the High-Performance Computing Center at UFRN (NPAD/UFRN). Each analysis step was executed separately, and the complete repository can be accessed at github.com/dalmolingroup/metastasisevolution.

### Ethical Issues

This study was based exclusively on computational and publicly available genomic datasets. No experiments involving live vertebrates or human subjects were conducted.

## Results

### Evolutionary network of biological processes involved in the initial stages of the metastatic cascade

**Figure 2-:**
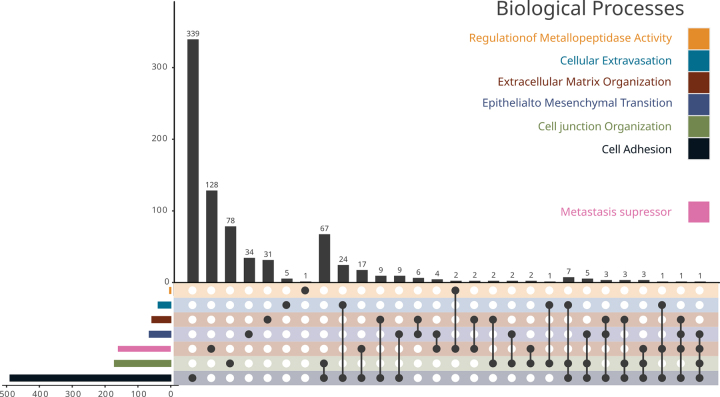
Metastasis-related genes are predominantly pleiotropic, participating in multiple biological processes rather than isolated pathways. The UpSet plot illustrates the distribution and extensive intersection of orthologs across the six fundamental biological processes associated with the early stages of metastasis and MSGs.

Our analysis began with the identification of genes associated with six biological processes fundamental to the early metastatic cascade: cell adhesion, ECM organization, regulation of metallopeptidase activity, cell junction organization, cellular extravasation, and EMT. By leveraging COG annotations from the STRING database, this initial set was filtered to a final dataset comprising 787 protein-coding genes distributed across 473 OGs. This set of genes had the vast majority participating in multiple processes rather than being exclusive to a single one (Figure 2). To address this pleiotropy, we conducted a parallel analysis restricting the dataset to strictly “exclusive orthologs” (Figure S1), which confirmed no major differences at evolutionary roots. Subsequently, we performed an evolutionary rooting analysis on each of these 473 OGs using the GeneBridge R package. This analysis aimed to identify the most ancient, vertically inherited archetype for each OG throughout evolution.

**Figure 3 -:**
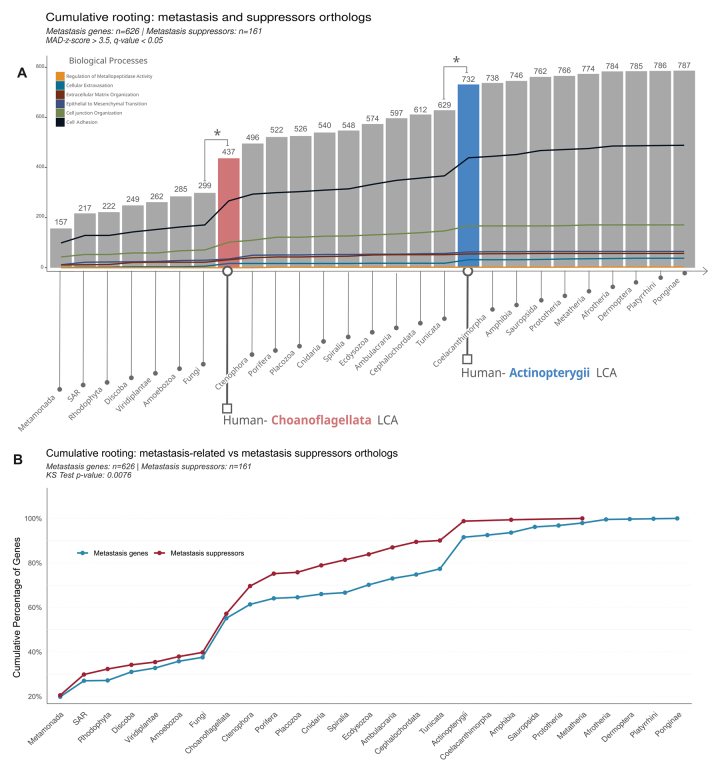
Divergent evolutionary origins and emergence peaks of metastasis-related orthologs and suppressors. (A) Evolutionary emergence peaks displaying the number of orthologs associated with early metastasis rooted in specific clades. Significant outliers representing major bursts of ortholog emergence are highlighted (MAD-z-score > 3.5, q-value), notably peaking in the Human-Choanoflagellata and Human-Actinopterygii last common ancestors. (B) Cumulative rooting trajectories demonstrating the stark evolutionary divergence between metastasis-related orthologs and metastasis suppressors, revealing that the proportion of rooted suppressors follows a distinct and delayed evolutionary trajectory. Each line represents a distinct biological process or MSG group, identified by color.

Our evolutionary rooting analysis (Figure 3 A) revealed two major clades of significant ortholog emergence for metastasis-related processes. The clades corresponding to the last common ancestors of Human-Choanoflagellata and Human-Actinopterygii showed a statistically significant overrepresentation of rooted OGs compared to other clades (MAD-z-score > 3.5, q-value < 0.05; Table S1).

### Divergent evolutionary origins of metastasis-related orthologs and suppressors

**Figure 4-:**
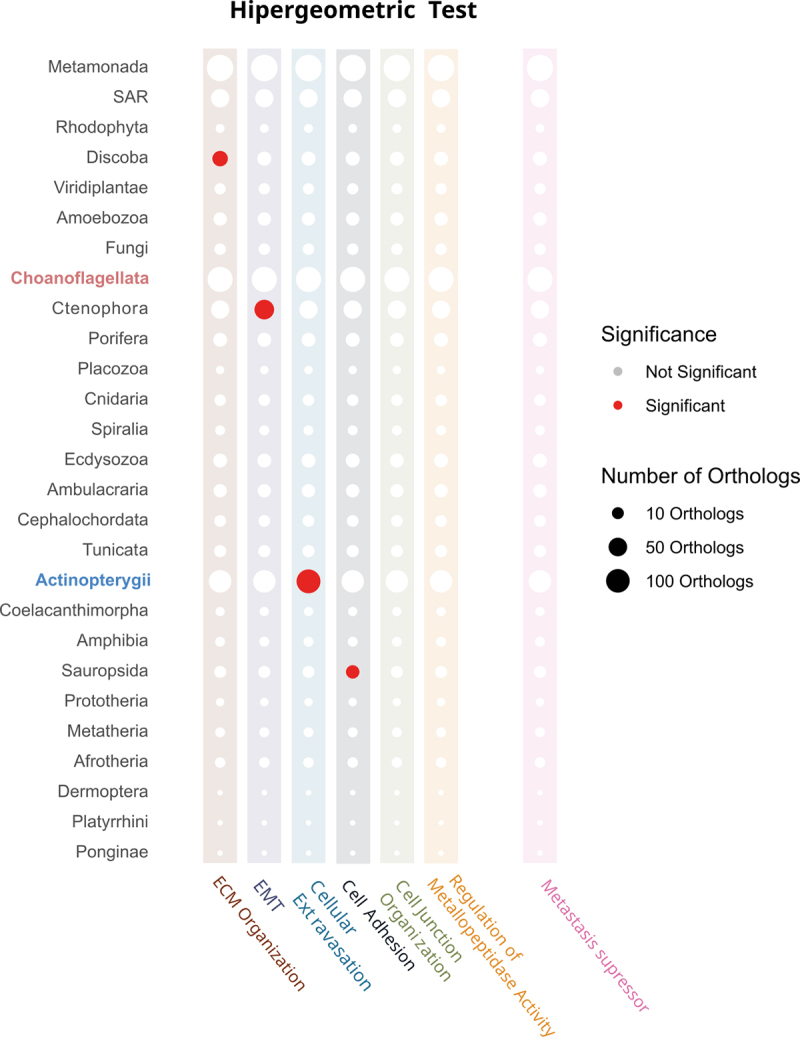
Divergent evolutionary origins of specific metastasis-related biological processes and MSGs. (A) The plot represents the hypergeometric functional enrichment of the six biological processes and MSGs across individual clades. Pathological metastatic programs demonstrate selective accumulation across distinct, ancient evolutionary junctures. Significance (q-value < 0.05) indicates whether a process is significantly over-represented among the orthologs rooted in a given clade.

To elucidate the evolutionary patterns of each biological process, we performed a hypergeometric test on the orthologs’ rooting distribution to identify which processes were statistically enriched within each clade of the phylogenetic tree (Figure 4). The enrichment analysis revealed a significant emergence in distinct evolutionary clades. Specifically, ECM organization was significantly enriched in the Discoba clade. Following this, at the base of the Metazoa, we observed a significant enrichment for EMT in Ctenophora. Later in evolution, cell adhesion emerged as significantly enriched in Sauropsida, and cellular extravasation was significantly enriched in Actinopterygii. In contrast, processes such as the regulation of metallopeptidase activity, as well as the overall emergence of isolated MSGs, did not show a statistically significant enrichment of rooting events in any individual clade.

However, by assessing the broader cumulative rooting trajectories (Figure 3 B ), we observed an evolutionary divergence between metastasis-related orthologs and metastasis suppressors. A comparison of the cumulative emergence curves demonstrated that the proportion of rooted suppressors followed a distinct evolutionary trajectory compared to the orthologs associated with metastasis (Kolmogorov-Smirnov test, p < 0.05).

### Functional enrichment of metastasis suppressor genes

**Figure 5 -:**
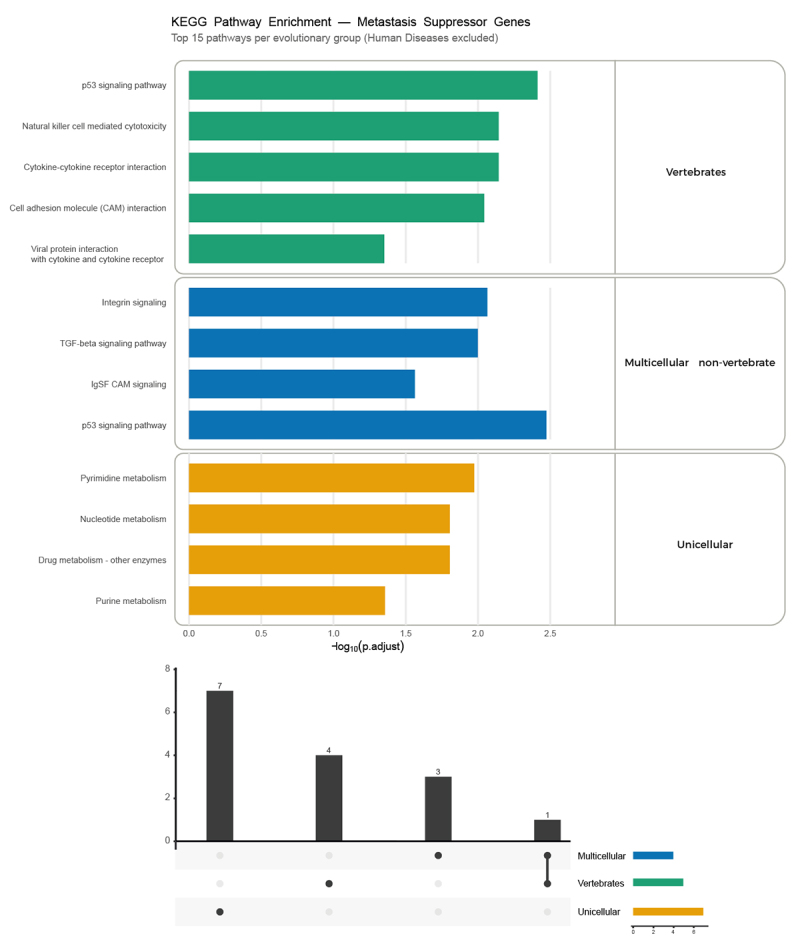
KEGG pathways transitions of MSG orthologs. This plot displays the KEGG enrichment results, presenting pathway representations corresponding to the distinct functional transitions of MSGs emerging across the Unicellular, Non-vertebrate Multicellular, and Vertebrate epochs.

**Figure 6 -:**
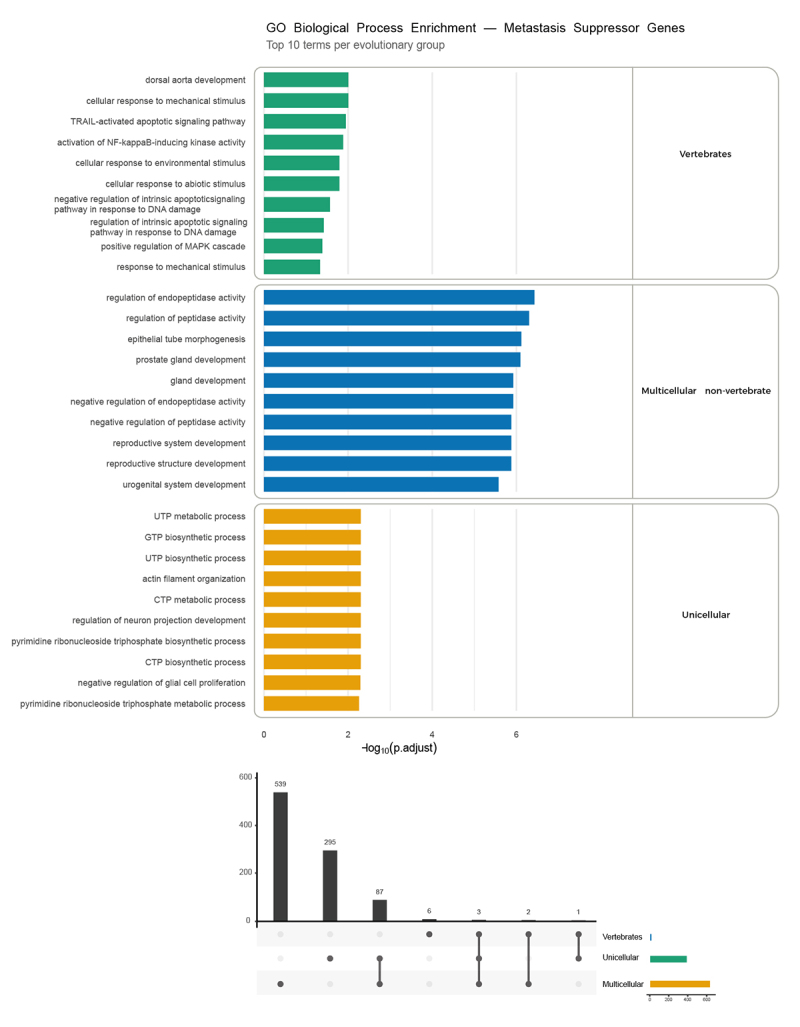
GO functional enrichment transitions of MSGs across major evolutionary epochs. This plot displays the GO enrichment results on enriched biological processes for MSGs originating during Unicellular, Non-vertebrate Multicellular, and Vertebrate stages. The functional profile highlights a distinct transition from basic metabolic pathways in early unicellular ancestors to the massive emergence of complex regulatory functions uniquely established in the Vertebrate lineage.

We conducted both isolated and cumulative functional enrichment analyses of MSGs (GO BP and KEGG). For this analysis, MSGs were grouped based on their Last Common Ancestor (Unicellular, Non-vertebrate Multicellular, or Vertebrates), as determined by the Bridge rooting analysis. We observed distinct functional transitions (Figures 5 and 6 , Figure S2). In unicellular ancestors, MSG orthologs were mostly restricted to basic metabolic pathways, such as nucleotide and pyrimidine metabolism. As evolution progressed into multicellularity, new suppressor functions emerged. However, it was within the Vertebrate lineage that we detected the emergence of complex regulatory metastasis suppression functions. This vertebrate-specific gain of function heavily emphasizes the control of mitosis, cell cycle regulation, apoptosis, and wound healing. In addition, we generated a comparative UpSet plot evaluating KEGG pathways shared between overall cancer pathways, MSGs, and metastasis genes (Figure S3).

## Discussion

The fundamental nature of metastasis is not characterized by the *de novo* invention of new biological capabilities by malignant cells, but rather by the pathological uncoupling of deeply conserved evolutionary layers. Our analysis of the evolutionary origins of early metastasis processes supports the SAM of cancer (Chen et al., 2015; Lineweaver et al., 2021), revealing an evolutionary divergence between the genetic machinery associated with metastasis (metastasis-related orthologs) and the machinery that halts it (suppressors).

The core toolkit for metastatic dissemination, including ECM organization, cellular motility (Cavalier-Smith and Chao, 2003; Sebé-Pedrós et al., 2013; Arthur et al., 2021), and EMT, is ancient. We observed significant enrichments of these metastasis-related modules in deeply rooted non-vertebrate clades, such as the Human-Discoba (aggregative multicellularity) (Sheikh et al., 2024) and Human-Ctenophora (early metazoan plasticity) LCAs (Ozbek et al., 2010; Sinkovics, 2015; Ye et al., 2024). At these ancient developmental stages, traits like unconstrained proliferation, loss of adhesion, and local invasion were not pathological; they were essential survival and morphogenetic mandates (Hynes, 2012; Lamouille et al., 2014). The ancient roots of these orthologs indicate that the structural and metabolic capacity for invasion, fueled by the pleiotropic nature of early genetic functional modules (Fares et al., 2020; Kiri and Ryba, 2024), is a robust, basal state ingrained within the eukaryotic genome.

However, metastatic dissemination within a complex host also co-opts more recent evolutionary innovations. Our results highlight that modules for cellular extravasation and cell adhesion were significantly enriched in the Actinopterygii (ray-finned fishes) and Sauropsida (reptiles and birds) LCAs, respectively. Extravasation, for instance, is physiologically dependent on vertebrate-specific systems such as a high-pressure closed vasculature and adaptive immunity, with many molecules involved in leukocyte trafficking being co-opted by cancer cells (Panara et al., 2024; Feuermann et al., 2025). In the context of the SAM, this reflects the sequential nature of neoplastic regression: as a tumor dedifferentiates, it first exploits the systemic transport programs of its host’s recent ancestors before ultimately reverting to basal metazoan tissue-disruption (EMT) and unicellular autonomy.

The critical insight of our study emerges when contrasting these ancient prometastatic roots against the evolutionary trajectory of MSGs. As demonstrated by the significant divergence in their cumulative rooting distributions, MSGs were not synchronously acquired alongside their metastasis-related counterparts. Instead, the capacity to actively suppress metastasis was a late-stage gain-of-function that peaked overwhelmingly at the emergence of the vertebrate lineage (Domazet-Lošo and Tautz, 2010).

This late surge in regulatory complexity is biologically intuitive. The advent of vertebrates introduced highly specialized tissues characterized by constant cellular turnover such as stratifying epithelia, gut linings, and a high-pressure, closed circulatory system capable of transporting immune cells worldwide. While continuous tissue renewal provided an immense evolutionary advantage, it created an inherently high risk of ubiquitous oncogenesis. To survive their own continuous mitosis and complex wound-healing demands, vertebrates were forced to evolve an elaborate network of physiological constraints including intricate apoptotic checkpoints, hormonal regulation of the cell cycle, and Natural Killer cell-mediated cytotoxicity. These are the exact functions identified in our cumulative MSG enrichment analysis for the Vertebrate clade. 

Therefore, viewing metastasis through the lens of the SAM provides an explanatory framework for its aggressiveness. Metastasis development is essentially the sequential deactivation of these evolutionary layers in reverse chronological order (Trigos et al., 2017). It begins as a localized failure of the most recently acquired vertebrate MSGs, the regulatory mechanisms responsible for maintaining tissue homeostasis and coordinating wound healing processes. Once these advanced, vertebrate-specific regulatory safeguards are compromised, the tumor is free to fall back upon the older, deeply robust toolkits of basal metazoa (e.g., EMT, collective migration) and, ultimately, unicellular autonomy (e.g., metabolic selfishness, uncontrolled proliferation). In this context, metastasis-associated genes do not acquire novel functions to enable dissemination; rather, their activity reflects the reactivation of evolutionarily conserved programs once released from vertebrate-specific regulatory constraints.

## Conclusion

This study examines the early stages of the metastatic cascade from an evolutionary perspective, shifting the analytical focus from exclusively mechanistic interpretations toward the SAM framework. By comparing the phyletic patterns of metastasis-related orthologs and suppressors, we demonstrate that metastasis is probably characterized by an evolutionary mismatch. The biological processes that enable cancer to spread, ECM remodeling, EMT, and basic motility, are ancient innovations that emerged with the origin of multicellularity. In contrast, metastasis suppressor genes appear to have arisen later in vertebrate evolution, likely in association with the need to regulate context-dependent gains-of-function, including sustained tissue renewal.

Consequently, metastasis should not be regarded as a novel set of traits invented by modern tumors. Instead, it is the pathological reversal of evolutionary history, the catastrophic failure of recently evolved vertebrate constraints that inevitably unleashes the ancient, dormant survival programs of earlier ancestors. Recognizing metastasis as a process of serial atavistic regression provides a cohesive theoretical framework that could influence how we approach the targeting of these deeply entrenched, basal biological drives.

## Supplementary material

The following online material is available for this article:

Figure S1 - Rooting distribution analysis of strictly exclusive metastasis-related orthologs.

Figure S2 - Cumulative functional enrichment heatmap across evolutionary clades.

Figure S3 - Comparative overlap of functional pathways among KEGG hsa:05200 cancer pathways, Metastasis Suppressor Genes, and metastasis-related genes.

Table S1 - Statistical significance of orthologous emergence across clades.

## Data Availability

 All data supporting the findings of this study are included within the article and its supplementary materials. The code used for the analyses is available at GitHub (https://github.com/dalmolingroup/metastasisevolution).
