# Supplementary material for: Reconstruction of the evolutionary landscape of biological processes involved in the early stages of the metastatic cascade
Source: Genet Mol Biol. 2026 Jun 29;49(Suppl 2):e20250197. doi: 10.1590/1678-4685-GMB-2025-0197 (PMC13329720; doi:10.1590/1678-4685-GMB-2025-0197)
Supplement: Figure S1 - [file 1415-4757-GMB-49-s2-e20250197-s1.pdf]

Genetics and Molecular Biology
https://doi.org/10.1590/1678-4685-GMB-2025-0197

Supplementary Material to "Reconstruction of the evolutionary landscape of biological processes involved in the early stages of the metastatic cascade"

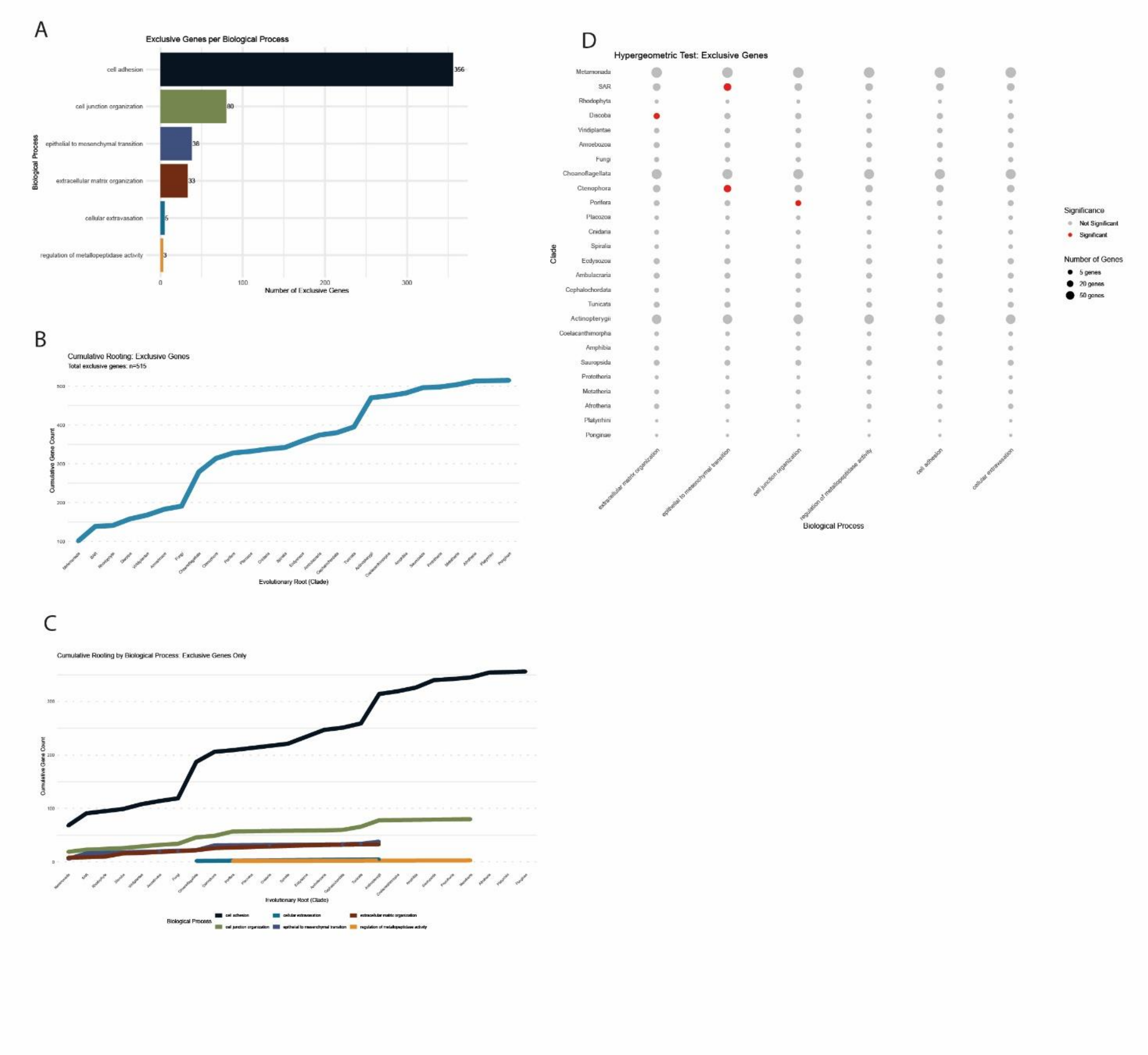

Figure S1 - Rooting distribution analysis of strictly exclusive metastasis-related orthologs.
