# Supplementary material for: Reconstruction of the evolutionary landscape of biological processes involved in the early stages of the metastatic cascade
Source: Genet Mol Biol. 2026 Jun 29;49(Suppl 2):e20250197. doi: 10.1590/1678-4685-GMB-2025-0197 (PMC13329720; doi:10.1590/1678-4685-GMB-2025-0197)
Supplement: Table S1 - [file 1415-4757-GMB-49-s2-e20250197-s4.pdf]

Genetics and Molecular Biology

https://doi.org/10.1590/1678-4685-GMB-2025-0197

Supplementary Material to “Reconstruction of the evolutionary landscape of biological processes involved in the early stages of the metastatic cascade”

Table S1 - Statistical significance of orthologous emergence across clades. The table details the modified Z-scores (MAD) and corresponding q-values for the evolutionary rooting distribution of metastasis-related orthologs.

root	clade_name	n	modified_z_scores	p_value	p_adjusted	cumulative
36	SAR	60	3.447	0.0005	0.004	217
35	Rhodophyta	5	-0.674	0.499	0.866	222
34	Discoba	27	0.974	0.329	0.866	249
33	Viridiplantae	13	-0.074	0.940	1	262
32	Amoebozoa	23	0.674	0.499	0.866	285
31	Fungi	14	0	1	1	299
30	Choanoflagellata	138	9.293	1.498E-20	3.89E-19	437
29	Ctenophora	59	3.372	0.0007	0.004	496
28	Porifera	26	0.899	0.368	0.866	522
27	Placozoa	4	-0.749	0.453	0.866	526
26	Cnidaria	14	0	1	1	540
25	Spiralia	8	-0.449	0.652	0.893	548
24	Ecdysozoa	26	0.899	0.368	0.866	574
23	Ambulacraria	23	0.674	0.499	0.866	597
22	Cephalochordata	15	0.074	0.940	1	612
21	Tunicata	17	0.224	0.822	1	629
20	Actinopterygii	103	6.670	2.557E-11	3.324E-10	732
19	Coelacanthimorpha	6	-0.599	0.548	0.891	738
18	Amphibia	8	-0.449	0.652	0.893	746
17	Sauropsida	16	0.149	0.880	1	762
16	Prototheria	4	-0.749	0.453	0.866	766
15	Metatheria	8	-0.449	0.652	0.893	774
14	Afrotheria	10	-0.299	0.764	0.993	784
10	Dermoptera	1	-0.974	0.329	0.866	785
7	Platyrrhini	1	-0.974	0.329	0.866	786
4	Ponginae	1	-0.974	0.329	0.866	787
